# Building capacities of Auxiliary Nurse Midwives (ANMs) through a complementary mix of directed and self-directed skill-based learning—A case study in Pune District, Western India

**DOI:** 10.1186/s12960-020-00485-9

**Published:** 2020-06-17

**Authors:** Shilpa Karvande, Vidula Purohit, Somasundari Somla Gopalakrishnan, B. Subha Sri, Matthews Mathai, Nerges Mistry

**Affiliations:** 1 Foundation for Research in Community Health, Pune, India; 2 Liverpool School of Tropical Medicine-Centre for Maternal and Newborn Health, Liverpool, UK

**Keywords:** Maternal and child health, Capacity building, Auxiliary nurse midwives, In-service training, Skill-based learning, Self-directed learning, India

## Abstract

**Abstract:**

Auxiliary nurse midwives (ANMs) play a pivotal role in provision of maternal and newborn health at primary level in India. Effective in-service training is crucial for upgrading their knowledge and skills for providing appropriate healthcare services. This paper aims at assessing the effectiveness of a complementary mix of directed and self-directed learning approaches for building essential maternal and newborn health-related skills of ANMs in rural Pune District, India.

**Methods:**

During directed learning, the master trainers trained ANMs through interactive lectures and skill demonstrations. Improvement and retention of knowledge and skills and feedback were assessed quantitatively using descriptive statistics. Significant differences at the 0.05 level using the Kruskal-Wallis test were analysed to compare improvement across age, years of experience, and previous training received. The self-directed learning approach fulfilled their learning needs through skills mall, exposure visits, newsletter, and participation in conference. Qualitative data were analysed thematically for perspectives and experiences of stakeholders. The Kirkpatrick model was used for evaluating the results.

**Results:**

Directed and self-directed learning was availed by 348 and 125 rural ANMs, respectively. Through the directed learning, ANMs improved their clinical skills like maternal and newborn resuscitation and eclampsia management. Less work experience showed relatively higher improvement in skills, but not in knowledge. 56.6% ANMs either improved or retained their immediate post-training scores after 3 months.

Self-directed learning helped them for experience sharing, problem-solving, active engagement through skill demonstrations, and formal presentations. The conducive learning environment helped in reinforcement of knowledge and skills and in building confidence. This intervention could evaluate application of skills into practice to a limited extent.

**Conclusions:**

In India, there are some ongoing initiatives for building skills of the ANMs like skilled birth attendance and training in skills lab. However, such a complementary mix of skill-based ‘directed’ and ‘self-directed’ learning approaches could be a plausible model for building capacities of health workforce. In view of the transforming healthcare delivery system in India and the significant responsibility that rests on the shoulder of ANMs, a transponder mechanism to implement skill building exercises at regular intervals through such innovative approaches should be a priority.

## Background

India has made commitments for universal health coverage and strengthening primary healthcare through the National Health Policy [1] and Declaration of Astana [2]. A skilled health workforce is one of the key requirements for achieving this commitment [3]. India has more than 200 000 auxiliary nurse midwives (ANMs)^1^ [4], one of the most important frontline health workforces of the Indian public health system. These ANMs play a pivotal role in primary healthcare delivery, particularly in the provision of maternal and newborn health (MNH) services.

Studies in Asian countries [5, 6], including India [7, 8], have identified deficits among health providers in MNH-related skills, especially midwifery skills. The evidence confirms an urgent need to strengthen skills of health workers, especially through in-service training [9]. India has challenges in building capacities of the health workforce because of inadequate training resources [10] and lack of need-based training [3]. Pre-service education and in-service training of ANMs in India have been adversely affected by lack of competent faculty, teaching aids, and funds for practical training, leading to sub-optimal outcomes from the training for service delivery [7, 11]. While in-service training courses aim to update ANMs on current best practices and skills, these are mostly in the form of a short orientation with minimal hands-on learning [12]. The exceptions are the recent initiatives such as skills training centres at national- and regional-level skills lab targeting the improvement of the quality of essential obstetric and newborn care [13]. The national training strategy formulated under the National Rural Health Mission envisages integrated training with the focus on skill upgradation [14]. In addition to a skills-focused approach, the principles of adult learning with a focus on self-directed approach have also been discussed in the realm of medical education [15]. Furthermore, studies have appreciated learner centric approaches and the importance of engaging participants in training [16, 17]. However, the extent of application of these principles and learner centric approaches in the planning and execution of any in-service training of health providers in India is unclear.

In this context, we share experiences from the capacity building of ANMs through skill-based directed and self-directed learning and the effectiveness of this mixed-learning approach. This paper is based on a pilot intervention undertaken for building MNH skills of ANMs in rural Pune District from January 2017 to January 2019 (2 years). This intervention applied directed and self-directed learning approaches respectively for the initial delivery and reinforcement of knowledge and skills of ANMs. This was approved and supported by the Public Health Department of the Government of Maharashtra, India.

## Methods

### Setting

Pune, Maharashtra, with a population of 9 429 408 [18] is the fourth most populous district in India. Around 39% of the population in Pune District lives in 13 rural sub-districts and is offered primary healthcare, including MNH care mainly through public health facilities—sub-centres (SCs = 539) and primary health centres (PHCs = 96) [19]. There are 703 ANMs posted across these health facilities—528 in regular and 175 in contractual positions.

### Development of learning materials

The development of learning materials for the intervention was guided by a baseline assessment of learning needs from ANMs and other key stakeholders at district, state, and national levels. In addition, current best practices for MNH care at primary level, international experiences in teaching methods, and alignment with state and national clinical protocols were referred. The learning materials included interactive presentation slides and guides for drill-based training of technical skills required in MNH care. The content included antenatal, intranatal, postnatal, and newborn care, life-saving skills such as maternal and newborn resuscitation, triaging of patients, management of eclampsia, obstetric haemorrhage, sepsis, effective communication during referral of patients, and additional topics such as maternal mental health, respectful maternity care, and importance of data and reporting. Mannequins were used for skill demonstrations. The learning materials were produced in both English and Marathi and were reviewed and approved by the Public Health Department of the Government of Maharashtra.

### Creating a group of trainers for the district

The intervention was based on a Training of Trainers (TOT) model. Medical doctors and nurses (*n* = 38) recommended by Pune District’s government authorities based on their previous training experience and willingness to participate were trained as master trainers in two batches of 6 days each by seven international technical experts. The first 4 days of the course focused on standardization of essential knowledge and technical skills through repetitive drills and comprehensive pre- and post-training assessment of knowledge and skills.

The additional 2 days focused on assessment of teaching skills such as delivery of content with clarity, capacity to engage participants, voice modulation, and adherence to training material, followed by inputs in pedagogical skills for interactive lectures, simulation-based practical demonstrations, and active engagement of all participants. Based on the technical and teaching assessment, 15 ‘best master trainers’ (six medical doctors and nine nurses) were selected for conducting subsequent directed and self-directed learning sessions of ANMs. To ensure the expected quality in the training, the first batch of ANM training was conducted by the selected ‘best master trainers’ in the presence of the international technical experts. The experts provided constructive feedback and necessary support to the trainers at regular intervals through quality assessment visits and refresher training programmes throughout the downstream intervention.

### Directed learning of ANMs

Directed learning of ANMs involved 4 days of training including 22 short interactive presentations, 24 skill demonstrations, and a role-play. ANMs were assessed for knowledge and clinical skills immediately before and after the training by the master trainers, using tools developed during this intervention. The knowledge assessment questionnaire included 30 dichotomous questions with true/false options; each correct answer scored one mark. The skills—maternal resuscitation, newborn resuscitation, eclampsia management, and use of partograph—were assessed by the master trainers using checklists and demonstration on mannequins (for the first three skills), with scores given for correctness and sequence of steps. A scenario-based exercise was used to assess plotting and interpretation of the partograph. The maximum scores were 30 for knowledge; 20 each for maternal resuscitation, newborn resuscitation, and management of eclampsia; and 15 for use of partograph.

At the end of each training session, the participating ANMs were asked to provide feedback about the quality of training and its relevance in routine practice. They rated each session on a scale of ‘1–5’ with ‘1’ having the least value and ‘5’ being the most effective, and provided supportive comments for their ratings.

Three months after the training, the master trainers further assessed 50 randomly selected ANMs at their workplaces for retention of knowledge and skills using the same assessment tools.

### Self-directed learning of ANMs

Self-directed learning of ANMs included four learning platforms—learning by ‘doing’, ‘seeing’, ‘writing’, and ‘interacting’. As per the conventional definition of self-directed learning [20], learners have more involvement in deciding the learning needs, methods, and appropriate resources. During the present project, the ANMs decided the learning needs and volunteered to participate in these activities.

#### Learning by ‘doing’

‘Skills mall’, a 1-day event for reinforcing the technical skills of ANMs, was organized on a Sunday to ensure maximum participation and was managed by the master trainers. Information about this event was circulated to all ANMs in the district. Interested ANMs voluntarily pre-registered for learning two preferred skills such as management of eclampsia, obstetric haemorrhage, and newborn resuscitation. Additionally, ‘soft skills’ such as communication skills and stress management for ANMs were included as per the requests received and were facilitated by external subject experts. Three such events were organized during the project—two at the district headquarters and one at the sub-district level based on the requests of the ANMs.

#### Learning by ‘writing’

Three ‘ANM Varta’ [ANM News] biannual newsletters were published with the themes ‘Quality in healthcare—role of ANMs’, ‘In-service training programmes for ANMs—experiences and way forward’, and ‘Experiences related to this ANM skill building project’. An appeal to contribute an article in the newsletter was shared with the ANMs through various communication channels, and 18 ANMs voluntarily contributed articles. They interacted with the project staff for appropriateness of their article to the newsletter theme, structure of the article, and language revisions.

#### Learning by ‘seeing’

A group of ANMs had an opportunity of participating in an exposure visit to two institutions practising evidence-based MNH care: (1) Fernandez Hospital, Hyderabad—a private midwifery-led facility striving to seek cost-lowering methods of healthcare delivery complying with evidence-based protocols—and (2) Mahatma Gandhi Institute of Medical Sciences (MGIMS), Wardha, Maharashtra—a public sector institute attempting to integrate value-based medical education with accessible and affordable healthcare.

#### Learning by ‘interacting’

Participation in the 13th National Conference of Society of Midwives-India (SOMI) [21] was an opportunity for a group of ANMs to interact, present their work, and learn.

Nomination from the district health authorities based on the annual performance of the ANMs and volunteering by ANMs facilitated their selection for participation in the exposure visits and this conference.

### Data collection

Quantitative data were collected in the form of assessment scores immediately before and after the training and at 3 months’ interval using knowledge questionnaire and checklists for assessing the skills and feedback scores at the end of training session. Qualitative data were collected as (a) semi-structured feedback of participating ANMs for its value and relevance in terms of potential for application, (b) case narratives (using an interview guide) of 12 participating ANMs who had the opportunities to apply knowledge and skills learnt during this training to manage or refer high-risk or complicated maternal and/or newborn cases, and (c) key informant interviews (KIIs) (using an interview guide) of 15 health authorities from block, district, and state level for learning relevance and application of training into practice.

### Data management and analysis

Quantitative data collected through directed learning—demographics and assessment scores—were analysed using Microsoft Excel 2016 and SPSS Statistical Software (version 24). As the data were not normally distributed, median scores and interquartile ranges (IQRs) were reported. Improvement in the scores (median scores of immediate post-training assessment minus median scores of pre-training assessments) and retention of the scores (scores at 3 months of training minus immediate post-training assessment scores) for knowledge and skills were calculated. Significant differences at the 0.05 level using the Kruskal-Wallis test were analysed to compare improvement scores across different groups such as age, years of experience, and previous training received. Furthermore, ‘super achiever’ ANMs who had vast improvement in scores were operationally defined as ANMs having pre- and post-training assessment scores of less than 40% and more than 95% (to reflect correctness of all the critical steps in the demonstration) respectively across knowledge and four skills. Their profile was presented in terms of years of experience, age, and previous training received. During the retention assessment conducted at the workplace of participating ANMs, deviation of ± 5% from post-training scores was recorded as ‘same retention’. This margin of permissible error was considered against the disturbances affecting administration, observation, and step-wise demonstration of skills. Hence, the retention scores were categorized as (1) retained, (2) scored more, and (3) scored less.

Qualitative data collected through feedback of participating ANMs, their case narratives, and KIIs during evaluation were transcribed in the local language (Marathi), translated into English, and recorded in a word processor (Microsoft Word 2016). These data were analysed thematically for learning the perspectives and experiences of stakeholders regarding uniqueness, effectiveness, and relevance of the intervention.

The Kirkpatrick model [22] with four levels—reaction (satisfaction, engagement, and perceived relevance of training), learning (change in knowledge, skills, attitude, perceived confidence and commitment, etc.), behaviour (application of knowledge into practice and required drivers for the same), and results (health outcomes)—was applied for evaluation.

## Results

### Directed learning of ANMs

A total of 341 of 527 (65%) invited ANMs participated in the training conducted over 18 batches. They were drawn from 88 of the 96 PHCs across the 13 rural blocks of Pune District. Each training batch had at least four trainers, with a trainer-to-trainee ratio of 1:6.

#### Characteristics of participating ANMs

Of the participating ANMs, 70% ANMs were in the age group of 36–55, 72% ANMs had more than 10 years of work experience, and 33% ANMs had not received any in-service or refresher training related to maternal and child health in the previous 2 years (Table 1).

**Table 1:** Characteristics of participating ANMs

Sr. no.	Characteristics	Participating ANMs (***n*** = 341)	
		***n***	%
**1**	**Age (years)**		
	22–35	91	27
	36–55	239	70
	≥ 56	11	3
**2**	**Experience (years)**		
	≤ 10	97	28
	11–20	100	29
	21–30	80	24
	>30	64	19
**3**	**MCH-related training received in preceding 2 years**	230	67

#### Pre- and immediate post-training assessment of knowledge and skills of participating ANMs

The scores for knowledge and skills improved after receiving the training. The median scores for improvement were particularly high for the three life-saving skills: maternal resuscitation 70 (IQR 55–80), newborn resuscitation 60 (IQR 45–75), and management of eclampsia 60 (IQR 50–70), but less for knowledge 13.3 (IQR 10.0–23.3) and use of partograph 26.6 (IQR 6.6–60.0) (Table 2).

**Table 2:** Knowledge and skill scores—pre- and post-training assessment scores (%)

Assessment	Participating ANMs (***n***)	Pre-training median scores (%)	IQR	Post-training median scores (%)	IQR	Change in median score (%)	IQR
**Knowledge**	311	60.0	53.3–63.3	73.3	70.0–80.0	13.3	10.0–23.3
**Maternal resuscitation**	224	20.0	10.0–30.0	90.0	80.0–95.0	70.0	55.0–80.0
**Newborn resuscitation**	225	15.0	10.0–30.0	85.0	70.0–90.0	60.0	45.0–75.0
**Management of eclampsia**	216	25.0	20.0–35.0	90.0	80.0–95.0	60.0	50.0–70.0
**Use of partograph**	230	26.6	20.0–66.6	80.0	60.0–93.3	26.6	6.6–60.0

*IQR* interquartile range

ANMs with ‘10 or less years of work experience’ and of younger age group (age group 20–35 years) showed relatively higher improvement across all the assessment with significant improvement for maternal resuscitation (*P* value 0.033 and 0.045) and newborn resuscitation (*P* value 0.009 and 0.003). Previous training received by the ANMs did not show any significant relation with the improvement in either knowledge or skills of the ANMS (Table 3).

**Table 3:** Effect of age groups and work experience of participating ANMs on improvement in knowledge and skills

Improvement	***n***	Age groups (in years)					Work experience (years)					
		Mean rank*			Chi-square	***P*** value	Mean rank*				Chi-square	***P*** value
		20–35	36–55	≥ 56			≤ 10	11–20	21–30	> 30		
Knowledge	311	172.7	149.8	142.9	4.274	0.118	169.2	151.6	152.4	146.5	2.942	0.401
Maternal resuscitation	224	125.4	108.9	72.3	6.216	**0.045**	125.4	106.8	119.3	90.2	8.757	**0.033**
Newborn resuscitation	225	132.0	107.2	64.4	11.368	**0.003**	132	111.4	106.7	90.4	11.575	**0.009**
Management of eclampsia	216	115.9	105.8	96.8	1.478	0.478	114.3	103.0	100.7	116.9	2.535	0.469
Use of partograph	230	115.8	114.4	134.8	0.725	0.478	116.3	113.2	114.5	118.8	0.198	0.978

*The Kruskal-Wallis test was used to compare pre-test to post-test improvement in knowledge and skills across age groups and work experience

#### Super achiever ANMs

Further analysis showed that some ANMs were super achievers (scoring < 40% in pre- and > 95% in post-training assessment) in four skills, more in maternal resuscitations (*n* = 80) and less in use of partograph (*n* = 13). The ANMs with 10 or less years of work experience predominantly scored better in each assessment (Table 4), of which four ANMs could attain ‘super achiever’ status across all the four life-saving skills.

**Table 4:** Distribution of super achievers as per age groups and work experience

Test	Number of ANMs participated in the test	Number of super achiever ANMs*	Age group			Work experience			
			22–35 years	36–55 years	56 years and more	10 years or less	11–20 years	21–30 years	30 years and more
Knowledge	311	0 (0%)	–	–	–	–	–	–	–
Maternal resuscitation	224	80 (36%)	37	43	0	39	21	15	5
Newborn resuscitation	225	44 (20%)	19	25	0	21	10	8	5
Management of eclampsia	216	63 (29%)	26	37	0	28	18	8	9
Use of partograph	230	13 (6%)	5	7	1	6	4	1	2

#### Retention of knowledge and skills at 3 months post-training

Fifty participating ANMs were randomly selected for assessing retention of knowledge and skills 3 months after the training. Of these, 56.6% (a combined average) ANMs either scored more or retained their immediate post-training scores for knowledge and four skills. The percentage of ANMs who retained or scored more was highest for use of partograph (64%) and lowest for newborn resuscitation (44%) (Table 5).

**Table 5:** Participating ANMs’ performance in retention test compared to the post-training assessment

Comparative performance during assessment of retention	Number of participating ANMs (%)				
	Knowledge test (***n*** = 50)	Maternal resuscitation (***n*** = 50)	Newborn resuscitation (***n*** = 50)	Management of eclampsia (***n*** = 44)	Use of partograph (***n*** = 50)
Retained* same scores	38	38	30	45	18
Scored more**	22	18	14	14	46
Scored less***	40	44	56	41	36

*Retained—retained same or deviation of ± 5% from post-training scores

**Scored more—> 5% of their immediate post-training scores

***Scored less—< 5% of their immediate post-training scores

#### Feedback of participating ANMs

The feedback received from the participating ANMs at the end of each training session had an average rating of 4.86 (4.63–4.96) for quality and 4.85 (4.64–4.96) for relevance on a scale of ‘5’. The three major reasons reported to justify their ratings were as follows: need-based training, emphasis on practical demonstrations, and conducive learning environment. They mentioned that the training programme matched their needs, with the thrust on case management at primary level and/or appropriate referrals. Secondly, they valued the emphasis on practical demonstration by the trainers and then by the trainees, helping everyone to practise the skills in a step-wise manner, which was a first of its kind for most of them. Thirdly, the conducive learning environment in terms of punctuality; competency, enthusiasm, and respectful behaviour of trainers; and easy to understand training methods were most appreciated. The most common feedback was ‘This training has reduced our fear of managing complicated or high-risk cases and we will certainly try to stabilize the patient at our level before referring her to a higher centre’.

### Applying learning to practice

Qualitative data collected towards the end of the intervention, through case narratives of ANMs and key informant interviews of key stakeholders, provided information about applying the learning of this intervention to practice in three areas—early and correct diagnosis, correct treatment, and increased confidence (Table 6).

**Table 6:** Examples highlighting application of learning to practice

Sr. no.	Areas	Change/improvement	Example/s	Quote
1	Diagnosis	Early and correct diagnosis	1. Need for suction among newborns 2. Identification of high risks during labour	‘Before this training, my ANMs would call me during nights for deliveries, especially plotting partograph. But now, they plot the partographs and only if there is any life-threatening condition, they call me to take my opinion’—Medical Officer PHC
2	Case management	Correct management	1. Routine ANC care 2. Newborn resuscitation	‘I first give a left lateral position to every pregnant woman; I do not forget it.’—ANM ‘I conducted a normal [vaginal] delivery. The baby did not cry. I immediately resuscitated the baby and the baby cried. Earlier at such times, we used to be scared. This time I could handle the case confidently.’—ANM
3	Confidence during case management	Increased confidence for giving drug/s	1. Use of magnesium sulphate for eclampsia management	‘I recently received feedback from the field that two ANMs gave Magsulf and managed the case. We wanted to promote this they used to be anxious about it and we couldn’t do it and now it is happening.’—District health official

### Self-directed learning of participating ANMs

Overall, 125 ANMs participated in self-directed learning activities. The findings below describe the uniqueness or innovative nature and perceived relevance of each activity into routine practice.

#### Learning by ‘doing’ through skills mall

Attending the skills mall provided a platform for ANMs to upgrade their skills, share their experiences of dealing with complications, and discuss possible solutions with the trainers. During the skills malls, though the ANMs (*n* = 59 of which 57 attended directed learning) had pre-registered only to learn two skills, all of them participated and practised three or more skills of their own choice.

‘We enjoyed the mall because of the interactive sessions and encouraging environment. The best thing was… whatever was demonstrated to us, we could demonstrate it back to the trainers.’—Participant ANM

‘It [skills mall] should be repeated every 6-12 months so that is becomes part of our regular training. It is very useful and necessary while working at a PHC level.’—Participant ANM

‘There are hardly any opportunities for our ANMs to learn such a mix of technical and non-technical skills e.g. communication skills. I really liked it.’—District health official

#### Learning by ‘seeing’—exposure visits

The ANMs (*n* = 15 of which 14 attended directed learning) valued the exposure visit as an opportunity for team-building, learning, interacting with seniors from other evidence-based health set-ups, and thus building their self-confidence.

‘For the first time we visited any such health facility, could freely discuss with the senior medical doctors and received a lot of respect. We really felt good.’—Participant ANM

After returning from the exposure visits, the ANMs presented views about application of observed practices in their routine practice to the district authorities.

‘We can implement the exercises during ANC, skills for management of eclampsia and respectful maternity care taught to us during the exposure visits, in our routine practice here.’—Participant ANM

#### Learning by ‘writing’ in the biannual newsletter

Through three biannual newsletters, the ANMs (*n* = 18 of which 17 attended directed learning) were motivated to think about a suitable article under the given themes. They discussed the related experiences and ideas with the project team and submitted the articles. For the first time, they went through such an academic and learning exercise.

‘Everyone cannot speak and share views effectively. I am more comfortable in writing than speaking, so the newsletter was a good way to share my views.’—Participant ANM

‘Writing an article based on this Project training was a reliving of the entire training. I could evaluate about what I could apply in my day-to-day practice and what could be the hurdles.’—Participant ANM

#### Learning by ‘interacting’ through participation in conference

During the participation in the conference, the ANMs (*n* = 4 of which two attended directed learning) listened to various presentations of midwifery experts and ANMs from other states and learned from their experiences.

‘For the first time we could interact with ANMs from other States and understood that there is such a platform like this conference where ANMs’ work can be appreciated. Otherwise we only work and submit reports.’—Participant ANM

The participants unanimously valued self-directed learning to complement other formal or directed learning and emphasized that such approaches should be institutionalized for revision of skills and building confidence of ANMs.

## Discussion

The paper discusses the value and challenges of a complementary mix of directed and self-directed learning as a potential approach for building skills of the public health workforce. Though a paradigm shift from ‘transfer of knowledge’ to ‘skill upgradation’ of all health providers during in-service training has been discussed about a decade ago [14], there is little published literature about subsequent attempts of revisiting training curricula and pedagogy. This project has provided initial evidence that a complementary mix of directed and self-directed learning can be effective for skill upgradation. This approach was specifically appreciated by the stakeholders due to its pedagogy focusing on a skills and drills approach, conducive learning environment, and adult learning principles [23] such as relevance, engaging participants, and being respectful [17].

For evaluation of effectiveness of this training model, the Kirkpatrick model of evaluation [22] was applied. The first level of ‘reaction’ and the second level of ‘learning’ were evaluated through feedback, and change in knowledge, skills, and perceived confidence, respectively. The third level, i.e. ‘behaviour’, to an extent, was evaluated through change in practices of the ANMs using the qualitative narratives. However, the fourth level of ‘results’, i.e. health outcomes, could not be evaluated within the short duration of the project.

The effectiveness of the directed learning with 4 days basic training was evident through improvement in knowledge, skills, and confidence of the participants. The ‘skills and drills’ platform proved to be more effective for clinical skills like improvement in management of eclampsia and maternal and newborn resuscitation rather than for the use of partograph which required more knowledge of graphing and numerical literacy. The nurse mentor programme in Bihar presented a similar pattern of improvement in the ability of ANMs across various clinical skills [8]. Perhaps for skills which were practised as close to natural clinical settings on mannequins, the acquisition as well as retention of skills was better. Knowledge which was tested by ‘True’ or ‘False’ questions attributed to factual memory recollection.

Less work experience of ANMs was associated with better absorption and demonstration of skills, but not with their knowledge. The ‘super achiever’ status was predominantly achieved by the less experienced ANMs. Multiple studies have documented conflicting effect of years of experience and age on knowledge and skills in the area of medical as well as paramedical trainin g[24–27]. Research on alternative training methods or strategies for elderly personnel would be desirable to achieve optimal increase in their essential skills.

The retention assessment after 3 months of training showed a drop in the scores compared to their immediate post-training scores for 43.6% ANMs; however, 64% ANMs either retained or improved their scores for the use of partograph. Limited occurrence and hence limited opportunities to apply the life-saving clinical skills such as maternal resuscitation and repeated use of other skills such as use of partograph in routine practice could be a possible reason behind this discrepancy in the retention scores.

The complementary booster dose of self-directed learning with a focus on voluntary participation was an effective platform for actively engaging participants. However, such boosters need to be repeated at regular intervals for retaining the gains as recommended by Ameh et al. [24] who emphasize on continuous learning through fire drills.

Careful selection, training, and assessment of trainers with necessary clinical skills and teaching ability are an absolute pre-requisite [28] for building the master trainers who can implement such a complementary mix of learning approaches. This study above all emphasizes on developing a sustainable pool of motivated and updated local trainers within the public health system who can invest time and efforts for imparting training at regular intervals. This will have a value especially in the context of evolving roles of health workforce including ANMs in the upcoming Ayushman Bharat Campaign^2^ [29].

## Limitations

During this study of 2 years, retention of knowledge and skills could not be assessed repetitively. The number of ANMs who were evaluated for learning application of skills in routine practice was also small. With a greater study duration, repeated assessment for retention of knowledge and skills as well as large-scale evaluation for application of skills into practice could be undertaken.

Since the participation of ANMs in the self-directed learning activities was voluntary and their number was low, we could not analyse this data quantitatively. A more systematic study with repeated self-directed learning activities with larger sample at regular interval can generate data for comparison.

Though the complementary mix of directed and self-directed learning approaches has been appreciated by the public health sector, real-life application of this approach within the public health system could not be assessed during the study period.

## Conclusion

In view of the transforming healthcare delivery system in India, regular hand-holding and capacity building including clinical mentoring for health providers would be necessary [29] for seamless provision of comprehensive primary healthcare. The demonstrated complementary mix of directed and self-directed learning with active participant engagement and assessment of learning outcomes can be potentially implemented as a skill building model. However, systemic investments in creating and sustaining competent, motivated, and updated trainers and conducive environments for absorption of learning can only warrant the successful implementation of this model for increasing capacities and competencies not only of ANMs in India but also of health providers globally. A significant responsibility rests on the shoulders of ANMs of reaching the desired goals of reduction in maternal and infant mortality and morbidity. Hence, we invite immediate attention of policymakers to develop a transponder mechanism to absorb and implement such complementary and effective approaches for capacity building of health providers including ANMs.

## Data Availability

The datasets used and/or analysed during the current study are available from the corresponding author on reasonable request.

## Abbreviations

ANMs: Auxiliary nurse midwives
IQR: Interquartile range
KIs: Key informants
MGIMS: Mahatma Gandhi Institute of Medical Sciences
MNH: Maternal and newborn health
PHCs: Primary health centres
PPIUCD: Postpartum intrauterine contraceptive device
SCs: Sub-centres
SOMI: Society of Midwives, India
TOT: Training of Trainers

## Footnotes

1 An ANM undergoes 2 years of nursing programme (ANM http://www.indiannursingcouncil.org/nursing-programs.asp?show=elig-crit) and is expected to work as a village-level multipurpose community health worker. Her role encompasses an array of programmes which include the maternal health, child health and family planning services, nutrition education, health education, collaborative service for improvement of environmental sanitation, immunization for control of communicable diseases, and treatment of minor ailments and first aid in emergencies and disasters. Besides these tasks, the ANM performs a supervisory role to the Accredited Social Health Activist (ASHA).

2 It was launched in 2018 with an aim for provision of comprehensive primary healthcare through differently staffed 150 000 Health and Wellness Centres (HWCs) with evolving roles of the health workforce including ANMs for provision of preventive, promotive, curative, rehabilitative, and comprehensive services to a population of 3000–5000.
